# Supplementary material for: SRSF3 promotes pluripotency through Nanog mRNA export and coordination of the pluripotency gene expression program
Source: eLife. 2018 May 9;7:e37419. doi: 10.7554/eLife.37419 (PMC5963917; doi:10.7554/eLife.37419)
Supplement: Figure 3—source data 4. [file elife-37419-fig3-data4.docx]

**Figure 3 – Source Data File 4. SRSF3 iCLIP mapping statistics.**

Significant binding sites, FDR<0.05.

|  | **EGFP-NLS** | **SRSF3-BAC** | **anti-SRSF3#** |
| --- | --- | --- | --- |
| Total reads | 5.6 x 10^6^ | 2.1 x 10^7^ | 8.2 x 10^6^ |
| Crosslink events | 5.5 x 10^4^ | 3.7 x 10^6^ | 9.3 x 10^4^ |
| Crosslink sites | 5.1 x 10^4^ | 3.3 x 10^6^ | 8.5 x 10^4^ |
| Significant crosslink events | 6.5 x 10^3^ | 4.3 x 10^5^ | 9.2 x 10^3^ |
| Significant binding sites | 3.2 x 10^3^ | 2.2 x 10^5^ | 5.2 x 10^3^ |
| Genes with >5 significant crosslink sites | 61 | 5,407 | 175 |

#single replicate experiment compared to three pooled experiments in EGF-NLS and SRSF3-BAC.
