# Supplementary material for: SRSF3 promotes pluripotency through Nanog mRNA export and coordination of the pluripotency gene expression program
Source: eLife. 2018 May 9;7:e37419. doi: 10.7554/eLife.37419 (PMC5963917; doi:10.7554/eLife.37419)
Supplement: Figure 6—source data 1. [file elife-37419-fig6-data1.docx]

**Figure 6 – Source Data 1. Read-wide mapping statistics.**

JunctionSeq was applied to analyse the RNA-seq data. All percentages are relative to the total read pairs and the filtering steps were consecutively applied from left to right. ^1^Percent reads that are not duplicates of another read pair and passed qorts filter. ^2^Percent reads that unambiguously aligned to a single gene. ^3^Percent reads that overlapped a splice junction. ^4^Percent reads that overlapped a novel splice junction.

| **Sample** | **Total**  **(10^6^)** | **Aligned (%)** | **Coherent (%)^1^** | **Unique**  **(%)^2^** | **Splice events (%)^3^** | **Novel events (%)^4^** |
| --- | --- | --- | --- | --- | --- | --- |
| D0-1 | 80.3 | 93.7 | 83.1 | 67.0 | 47.5 | 1.26 |
| D0-2 | 66.9 | 91.5 | 81.6 | 62.4 | 43.5 | 1.14 |
| D3-1 | 59.3 | 94.0 | 84.4 | 58.0 | 41.6 | 1.27 |
| D3-2 | 69.8 | 89.8 | 79.1 | 54.5 | 39.0 | 1.13 |
| D6-1 | 81.7 | 92.9 | 83.5 | 46.5 | 32.6 | 1.09 |
| D6-2 | 78.0 | 91.8 | 82.0 | 49.0 | 34.8 | 1.09 |
| D9-1 | 76.8 | 92.2 | 80.6 | 47.9 | 33.2 | 1.16 |
| D9-2 | 66.2 | 88.9 | 79.8 | 45.8 | 31.4 | 1.05 |
| D12-1 | 62.4 | 92.4 | 83.6 | 43.5 | 29.7 | 1.12 |
| D12-2 | 76.5 | 92.6 | 83.5 | 47.5 | 32.9 | 1.12 |
| iPSC-1 | 91.3M | 92.8 | 83.3 | 53.0 | 37.9 | 1.26 |
| iPSC-2 | 67.0M | 92.2 | 81.8 | 57.1 | 41.2 | 1.32 |
